# Development of Novel Antimicrobial Dental Composite Resin with Nano Cerium Oxide Fillers

**DOI:** 10.1155/2022/3912290

**Published:** 2022-04-12

**Authors:** Eldho Jijy Varghese, Dhanasekaran Sihivahanan, Kondas Vijay Venkatesh

**Affiliations:** Department of Conservative Dentistry and Endodontics, SRM Kattankulathur Dental College and Hospital, SRM Institute of Science and Technology, SRM Nagar Kattankulathur, Kanchipuram, Chennai 603203, Tamil Nadu, India

## Abstract

**Objectives:**

To assess the antibacterial efficacy of experimental dental composite resin with cerium oxide nanoparticles as fillers.

**Methods:**

The cerium oxide nanoparticles were prepared by the coprecipitation procedure. Synthesized 3wt% CeO_2_ nanoparticles were added to the composite resin as antibacterial filler. Experimental composite resin was manually prepared by adding ingredients. The resin matrix consisted of two mixed monomers, bisphenol A-glycidyl methacrylate and triethylene glycol dimethacrylate, diketone as the photo initiator, and N, N-dimethylaminoethyl methacrylate as a coinitiator. The antibacterial efficacy against *Streptococcus mutans*, *Streptococcus mitis*, *Streptococcus aureus*, and *Lactobacillus* spp. bacterial strains was tested using the microdilution method keeping commercially available 3M Filtek Z250 restorative composite as control.

**Results:**

The experimental dental composite demonstrated 99.503% efficacy against *Streptococcus mutans*, 99.441% efficacy against *Streptococcus mitis*, 99.416% efficacy against *Streptococcus aureus*, and 99.233% efficacy against *Lactobacillus* spp.

**Conclusion:**

Integrating cerium oxide nanoparticles as fillers into dental composite resin can be promising in terms of antibacterial activity, provided furthermore study has to be conducted to examine other properties. *Clinical Significance*. Previous studies attempted adding CeO_2_ nanoparticles into acrylic resins that showed improvement in mechanical properties, but literature is nil on the dental composite resin and cerium oxide nanoparticles. This study demonstrates the development of an experimental antibacterial dental composite resin that can resolve most of the problems related to secondary caries around dental composite restorations.

## 1. Introduction

Introduction of nanomaterials in the biomedical field makes it possible to overcome the difficulty in bacterial drug resistance attributed to their unique antibacterial mechanism. Additionally, a variety of metal and metal oxide-based nanomaterials have been fully integrated in antibacterial applications and achieved excellent performances. Among them, cerium and cerium oxide-based nanomaterials, which have lower toxicity, act as effective antibacterial agents owing to their unique functional mechanism against pathogens through the reversible conversion of the oxidation state between Ce(III) and Ce(IV). Thus, the current study idea highlights the application of cerium oxide nanoparticles in dentistry field, which is still a new area for research.

Dental caries is a biofilm-mediated, diet-dependent, multifactorial, noncommunicable, dynamic illness that causes net mineral loss in dental hard tissues. This multifactorial condition is caused by an imbalance between mineral loss (demineralization) and mineral gain (remineralization) in saliva. Microorganisms, substrate, host/teeth, and time are the primary variables that lead to the terminal stage of continuous mineral loss [1, 2].

Clinical restorative polymer materials are made up of a variety of materials with comparable primary chemical compositions, such as direct resin composite restorative, enamel-dentin adhesives, and dental primers (adhesion promoters). Previous studies have shown that resin-based restorative materials promote the formation of cariogenic biofilms [2]. The formation of cariogenic biofilm is due to the breakdown products from dental monomers such as bisphenol A-glycidyl dimethacrylate (BisGMA) and triethylene glycol dimethacrylate (TEGMA), which may change the metabolism of caries-related bacteria like *Streptococcus mutans* and increase their multiplication. [2].

The ensuing cariogenic biofilm causes destruction of the mineral structure of any intact, sealed, or repaired tooth surface where biofilm has developed and is regularly exposed to sugar. As a result, it has an impact on the initiation and progression of carious lesions not only in their first stages but also in their recurrence which leads to recurrent caries or caries around restorations (CARS) at the contact between the restoration and the prepared cavity. CARS rates for restorative polymer materials are quite high, at around 60%, and it has been identified as one of the leading causes of resin composite restoration failure. Given these obstacles, in order to improve the success rate of composite resin restoration, it is critical for the effective control of the formation of CARS [1, 2].

Metal and metal oxide nanoparticles have long been known to have antioxidant effects against microorganisms. The antioxidant effects are due to the movement of free electrons from the oxygen atoms present in the metal oxides to free radicals present on the microorganisms [3].

In an array of available metal oxides, the metal oxide nanoparticles of cubic fluorite type lanthanide series, such as cerium oxide (CeO_2_), have a considerable antibacterial effect and could be used to effectively remove pathogens. Furthermore, the CeO_2_ nanoparticles' (NP's) durability and delayed release of metal ions are crucial qualities that make them superior to other metal oxide nanoparticles. CeO_2_ has superior antioxidant property in comparison to other metal oxide nanoparticles because its transfer from the reduced to the oxidised state is reversible and the process can be restarted.

Literature search on CeO_2_ nanoparticles shows a promising impact against the oral microflora. Adding CeO_2_ nanoparticles to acrylic resins improved mechanical qualities in previous studies [22]. Literature is nil in the introduction of CeO_2_ nanoparticles as fillers in dental composite resin, in reducing the CARS and biofilm formation [4]. Hence the aim of this study was to evaluate the antibacterial efficacy of a novel experimental dental composite resin with cerium oxide nanoparticles as fillers. The null hypothesis of the study was dental composite resin with cerium oxide filler has no antibacterial efficacy against oral microflora.

## 2. Materials and Methods

### 2.1. Synthesis of Cerium Oxide Nanoparticles and Characterization

Synthesis was carried out in Department of Nanotechnology Research Centre (SRMIST, Kattankulathur). The CeO_2_ NPs were made using a process called coprecipitation. Initially, 0.04 g of sodium hydroxide of 0.1 M is produced in 0.5 ml of distilled water. In 20 ml of distilled water, 2.17 g of cerium (III) nitrate hexahydrate with a 0.5 M concentration is dissolved. Then, by continuously swirling, the NaOH solution was mixed with the precursor drop by drop. The solution was swirled at a steady speed for 20 minutes at room temperature. The mixture was centrifuged several times with distilled water and once with ethanol after it had been well combined. The resulting precipitate was then annealed in a laboratory oven for 20 minutes at 60°C to evaporate the sample's water content [5].

### 2.2. Synthesis of Experimental Composite Resin

Table 1 provides the ingredients and their concentrations used for the manufacture of the experimental composite resin. The matrix consisted of two mixed monomers, bisphenol A-glycidyl methacrylate (bisGMA) and triethylene glycol dimethacrylate (TEGDMA) (all purchased from Sigma-Aldrich, St. Louis, MO, USA). Additionally, Diketone (CQ, Sigma-Aldrich, St. Louis, MO, USA), as the photo initiator and N, N-dimethylaminoethyl methacrylate (DMAEMA) as a coinitiator (both Sigma-Aldrich, St. Louis, MO, USA).

### 2.3. Monomer Preparation

Bisphenol A-glycidyl methacrylate (BisGMA) was placed in a glass container and preheated to 50°C for 60 minutes followed by adding triethylene glycol dimethacrylate monomer (TEGDMA) to obtain a monomer mixture.

### 2.4. Silanization of Fillers

The reinforcing fillers such as amorphous silica (Evonic Industries, Essen, Germany) and to obtain the dental composite aluminium silicate fillers (Evonic Industries, Essen, Germany), cerium oxide nanoparticles were silanized. The fillers were compounded into a matrix in 50 mL glass Griffin form beakers at room temperature. The silane treatment of the fillers was prepared by the modified method which is known in art. Amorphous silica and aluminium silicate fillers are silanated by using alkoxy terminated silanating agents. A 1 vol.% of 3-methacryloxypropyltrimethoxysilane (Sigma-Aldrich, St. Louis city, USA) solution was prepared by using a preprepared solvent mixture of 90 vol.% ethanol and 10 vol.% deionized water. The pH of the solvent mixture was adjusted to 4 by 3.0 M acetic acid.

The silane solution was next stirred and allowed to hydrolyze (activate) for 1 h. The filler, silanating agent, and a ketonic solvent are taken in a glass vessel. The content is stirred for 5–8 h at 40–50°C; then, the solvent is decanted off, and the filler is dried at 105°C for 2-3 h and sieved before use in the composite. The fillers were dispersed by ultrasonication for 15 min to obtain a reaction mixture. Then, the reaction 5 mixture was stirred for 24 h at room temperature. After the silane grafting process, the reaction mixture was filtered and rinsed with absolute ethanol to remove physically adsorbed silanes.

The powder was dried overnight at room temperature and then dried at 60°C in an oven for 72 h to enhance the condensation of surface silanol molecules and to remove any remaining solvent. The surface elemental compositions of cerium oxide nanoparticles before and after silane grafting were examined by X-ray photoelectron spectroscopy, XPS (Perkin-Elmer PHI 5400, Waltham, MA, USA), with MgK radiation (h = 1253.6 eV).

The so obtained monomer mixture, silanized filler particles, diketone photoinitiator, DMAEMA coinitiator, UV stabilizer, and the inhibitor were weighed in sequence and added to and taken in a mortar and pestle. It was then mixed manually to get a mass and kept in the oven maintained at 40–50°C overnight. After 24 h of maintaining at 40–50°C, it was again mixed manually in the mortar for about an hour and kept back in the oven at 40–50°C [20]. This process was continued for 5–7 days or till desired consistency to obtain the dental composite [6].

### 2.5. Characterization of the Experimental Composite Resin

**Figure 1:**
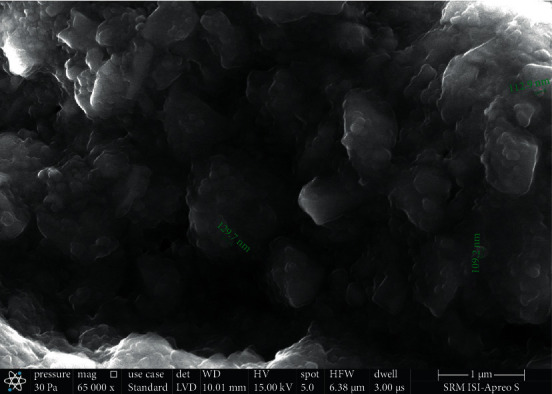
SEM image of experimental composite.

**Figure 2:**
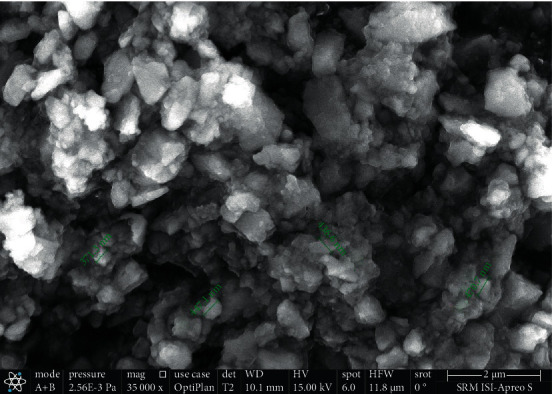
SEM image of control 3M Filtek Z250 composite.

Under high-vacuum circumstances, the 0.5 mg specimen was viewed using a Thermo Fisher Scientific Apreo S SEM with a resolution of 0.9 nm at 1 kV, landing energy of 20 eV to 30 keV, and a max beam current of 50 nA. The CeO_2_ NP filler has a homogeneous distribution with an average particle size of 117 nm, according to SEM examination. SEM imaging of 3M Filtek Z250 restorative composite was also carried out (Figures 1 and 2).

### 2.6. Evaluation of Antibacterial Efficacy

The analysis was performed in Tamilnadu Test House Private Limited (NABL accredited and drug control licensed). *Streptococcus mutans*, *Streptococcus mitis*, *Streptococcus aureus*, and *Lactobacillus* spp. bacterial strains were utilized [7].

Storage: these strains were always handled and preserved in microaerophilic conditions in an anaerobic jar, and they were cultivated in thioglycolate broth with a colorimetric indicator or blood supplemented agar, depending on the needs of the experiment.

Methodology: samples were randomly divided into two groups with five samples each. Group A included 3M Filtek Z250 restorative composite which was kept as control. Group B included the experimental composite resin. A bacterial initial inoculum (106 CFU/ml) freshly produced (also in thioglycolate broth) from a single colony was tested using the microdilution method (in a 96-well plate). The bacterial suspension was incubated with 2 g of the experimental composite resin for 18 hours at 37°C in an anaerobic jar that had been set up specifically for this purpose. Following this time, 10 L aliquots of the samples with less and no turbidity (as well as controls) were dropped on blood agar Petri dishes and incubated for 18 hours under same conditions. Finally, CFUs were counted. Gram staining was performed at the start and end of the experiment to ensure that strains were not contaminated.

## 3. Results

The analysis method used was according to EN 1276 : 2009. Data regarding microbial count CFU/ml, its log10 values, and its percentage of antibacterial efficacy of an experimental dental composite resin with cerium oxide nanoparticles as fillers and control material among 5 samples of each test organisms were entered into Microsoft Excel and analyzed using IBM SPSS Statistics for Windows, Version 20 (IBM Corp, Armonk, N.Y, USA). Data were investigated for normality using the Kolmogorov–Smirnov test, which showed that data were normally distributed. Descriptive statistics, i.e., mean, standard deviation, variance, and range were analyzed. The unpaired *t*-test was used to analyze the difference in microbial count and antibacterial efficacy between the experimental and control groups. The paired *t*-test was used to analyze the difference between initial and final time points in experimental and control groups. The level of significance was determined at *p* ≤ 0.05.

**Figure 3:**
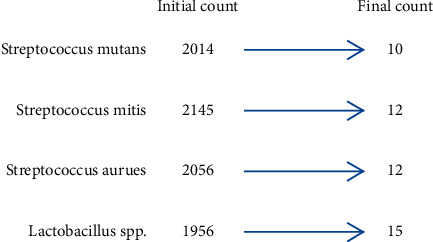
Bacterial count for experimental composite.

The experimental dental composite showed 99.503% efficacy against *Streptococcus mutans*, 99.441% efficacy against *Streptococcus mitis*, 99.416% efficacy against *Streptococcus aureus*, and 99.233% efficacy against *Lactobacillus* sppThe control groupshowed almostnil antibacterial efficacy. Tables 2–5 and Figure 3 show the initial and final count of colony forming bacterial units.

## 4. Discussion

Composite resin restoration influences the initiation and progression of carious lesions, not just in its primary development but also in its recurrence. Recurrent caries or caries around restorations (CARS) develop at the interface between the restoration and the prepared cavity as a result of restoration failure. The rates of CARS for restorative polymer materials are very high at approximately 60%, and it has been identified as one of the major reasons for the failure of resin composite restorations.

Aas and others (2008) in his research to determine the bacterial profile for dental caries have concluded that both *Streptococcus* and *Lactobacillus* species were the most commonly present bacteria in dental caries [7]. Hence, in this study, *Streptococcus mutans*, *Streptococcus mitis*, *Streptococcus aureus*, and *Lactobacillus* spp. were the experimental subjects for anaerobic bacterial analysis.

Based on previous studies by Dina and others, 2015, increasing the weight percent of nanoparticles above 3wt% resulted in decreased monomer radical mobility and decreased conversion with decrease in mechanical properties. Hence, in the current research, the optimum weight of CeO_2_ nanoparticles was kept at 3wt% [8].

The mechanism of antibacterial action of CeO_2_ nanoparticles is extensively studied, but literature on its application in dental composite resin is sparse. Through electrostatic contact, positively charged nanoparticles (NPs) are adsorbed onto the membranes of negatively charged bacteria. Gram-positive bacteria have a thicker layer of peptidoglycan. Because of the bacterial membrane blockage and electrostatic interaction, NPs stay on the surface of bacteria for a long time rather than being washed away. Rather than entering the membrane, it passes through it. The specialized ionic pumps are hampered by the membrane's viscosity. Eventually, this will have a significant impact on the transportation exchanges between the bacterial cell and fluid that causes bacterial growth to be disrupted [9].

After adsorbing on the bacterial cell's outer membrane, CeO_2_ can assault proteins. The released cerium ions could disrupt bacteria's electron flow and respiration by reacting with thiol groups (–SH) or being adsorbed onto transporters and/or porins, causing nutrient transfer to be hampered [10, 18, 19].

During the antibacterial process, oxidative stress is also a significant factor for CeO_2_. In general, oxidative stress is caused by the production of reactive oxygen species (ROS) in vivo, whereas ROS produced on the surface of bacterial membranes is caused by the reversible conversion of Ce(III) to Ce(IV) [11]. ROS can damage nucleic acids, proteins, polysaccharides, lipids, and other biological components, causing them to lose function and finally kill them [12].

An effective method for preventing enamel demineralization and appearance of cavitated lesions is the use of dental materials that are resistant to bacterial accumulation. Ideally, the material should suppress bacterial activity at tooth-restoration interphase. Nanotechnology represents a promising area of intense research in dentistry improving dental materials' antibacterial and mechanical properties. CeO_2_ NPs' cytotoxicity can be influenced by their size, shape, and surface charge. Due to larger specific surface areas, higher Ce3C levels, and higher cellular absorption, the smaller CeO_2_ NPs were more hazardous [13–23]. In this study, the average nanoparticle size was 117 nm.

Cerium and cerium oxide are antimicrobials in and of themselves, and compounds containing them have improved antimicrobial effects as well as other positive qualities such stimulating angiogenesis, osteogenesis, and wound healing. Researchers have made some progress in developing antibacterial drugs and biomedical materials, but it is far from sufficient. Limitations of this study include being an in vitro analysis. More animal tests and long-term effects observation are predicted to improve the application of cerium and cerium oxide-related antibacterial materials. More research into the cytotoxicity and mechanisms of new cerium and cerium oxide-based compounds is needed. This study explains the development of novel cerium oxide filler-based antimicrobial dental composite resin and its possibilities in dentistry.

## 5. Conclusion

Within the limitations of the study, it can be concluded that incorporating 3 wt% of cerium oxide nanoparticles as fillers into dental composite can be promising in terms of antibacterial efficacy, provided furthermore research has to be initiated to evaluate the mechanical and physical properties. This innovation can be an eye opener for future antibacterial composites.

## Figures and Tables

**Table 1:** Ingredients and their concentrations used for the manufacture of the experimental composite resin.

S. no.	Ingredient	Amount (%)
1	Bisphenol A-glycidyl methacrylate (BisGMA)	21
	(Aldrich make)	
2	Triethylene glycol dimethacrylate (TEGDMA) (Aldrich make)	10
3	Silanated amorphous silica (Evonik make)	9
4	Silanated aluminium silicate fillers (Evonik make)	55
5	Cerium oxide nanoparticles silanated	3
6	Diketone photo initiator (Aldrich make)	1
7	Dimethylaminoethyl acrylate coinitiator (DMAEMA) (Aldrich make)	0.5
8	UV stabilizer (Aldrich make)	0.25
9	Inhibitor (Aldrich make)	0.25

**Table 2:** Comparison of microbial count (CFU/ml) between the experimental group and control group for *Streptococcus mutans*.

Microbial count (CFU/ml)	Groups	*n*	Mean ± SD	Mean difference	Unpaired *t* value	df	*P* value
Initial	Dental composite resin with CeO_2_ nanoparticles	5	2013.80 ± 2.86	0.000	0.000	8	1.000
	Control	5	2013.80 ± 2.86				

Final	Dental composite resin with CeO_2_ nanoparticles	5	10.00 ± 1.58	−2000.40	−1330.64	8	0.000^*∗*^
	Control	5	2010.40 ± 2.96				

^*∗*^Statistically significant (*p* ≤ 0.05).

**Table 3:** Comparison of microbial count (CFU/ml) between the experimental group and control group for *Streptococcus mitis*.

Microbial count (CFU/ml)	Groups	*n*	Mean +SD	Mean difference	Unpaired *t* value	df	*P* value
Initial	Dental composite resin with CeO_2_ nanoparticles	5	2145.00 + 7.90	0.000	0.000	8	1.000
	Control	5	2145.00 + 7.90				

Final	Dental composite resin with CeO_2_ nanoparticles	5	12.00 + 1.58	−2182.20	−598.60	8	0.000^*∗*^
	Control	5	2140.20 + 7.79				

^*∗*^Statistically significant (*p* ≤ 0.05).

**Table 4:** Comparison of microbial count (CFU/ml) between the experimental group and control group for *Streptococcus aureus*.

Microbial count (CFU/ml)	Groups	*n*	Mean +SD	Mean difference	Unpaired *t* value	df	*P* value
Initial	Dental composite resin with CeO_2_ nanoparticles	5	2055.20 + 9.36	0.000	0.000	8	1.000
	Control	5	2055.20 + 9.36				

Final	Dental composite resin with CeO_2_ nanoparticles	5	11.80 + 2.86	−2034.60	−416.17	8	0.000^*∗*^
	Control	5	2046.40 + 10.54				

^*∗*^Statistically significant (*p* ≤ 0.05).

**Table 5:** Comparison of microbial count (CFU/ml) between the experimental group and control group for *Lactobacillus* sp.

Microbial count (CFU/ml)	Groups	*n*	Mean +SD	Mean difference	Unpaired *t* value	df	*P* value
Initial	Dental composite resin with CeO_2_ nanoparticles	5	1951.80 + 6.26	0.000	0.000	8	1.000
	Control	5	1951.80 + 6.26				

Final	Dental composite resin with CeO_2_ nanoparticles	5	14.60 + 2.07	−1928.80	−492.78	8	0.000^*∗*^
	Control	5	1943.40 + 8.50				

^*∗*^Statistically significant (*p* ≤ 0.05).

## Data Availability

The data used to support the findings of this study are available from the corresponding author upon request.
